# Metasurfaces Based on Phase-Change Material as a Reconfigurable Platform for Multifunctional Devices

**DOI:** 10.3390/ma10091046

**Published:** 2017-09-06

**Authors:** Niloufar Raeis-Hosseini, Junsuk Rho

**Affiliations:** 1 Department of Chemical Engineering, Pohang University of Science and Technology (POSTECH), Pohang 37673, Korea; niloufar@postech.ac.kr; 2 Department of Mechanical Engineering, Pohang University of Science and Technology (POSTECH), Pohang 37673, Korea

**Keywords:** metamaterial, chalcogenide, phase-change-material-based memory, germanium antimony telluride, tunable, phase transition

## Abstract

Integration of phase-change materials (PCMs) into electrical/optical circuits has initiated extensive innovation for applications of metamaterials (MMs) including rewritable optical data storage, metasurfaces, and optoelectronic devices. PCMs have been studied deeply due to their reversible phase transition, high endurance, switching speed, and data retention. Germanium-antimony-tellurium (GST) is a PCM that has amorphous and crystalline phases with distinct properties, is bistable and nonvolatile, and undergoes a reliable and reproducible phase transition in response to an optical or electrical stimulus; GST may therefore have applications in tunable photonic devices and optoelectronic circuits. In this progress article, we outline recent studies of GST and discuss its advantages and possible applications in reconfigurable metadevices. We also discuss outlooks for integration of GST in active nanophotonic metadevices.

## 1. Introduction

Metamaterials (MMs) are synthetic media that were developed by arranging components on a sub-wavelength scale; as a result, MMs have unnatural passive electromagnetic (EM) properties such as a negative refractive index (RI) or perfect absorption [1,2]. Significant research has been dedicated to the realization of MMs with resonances in high-RI and low-loss dielectrics. These efforts have provided methods to reduce the extensive ohmic losses that occur in plasmonic MMs at optical frequencies. MMs were initially conceived as way to achieve unprecedented EM properties in passive media [3]. However, reconfigurable nanostructures that use phase change materials (PCMs) [1,4] allow for the development of active and controllable MMs [5,6]. To design and fabricate a tunable photonic device that can be controlled by applying stimuli, a special material with the ability to change material phases is a fundamental requirement.

PCMs are nonvolatile, respond quickly to stimuli, and have reliable data retention. Their optical properties can be modified by applying electrical or thermal stimuli [7,8,9,10]. The main distinctive property of PCMs is that thermal and electrical activations or optical pulses can switch them from an amorphous phase to a crystalline phase [11]. The switch causes a large change in the RI [12], so PCMs are excellent for applications such as rewritable DVDs [13], plasmonics [14], photonics [15], and MMs [16].

Nanoelectronic PCM-based random access memories (PCRAMs) [13] and optical data storages benefit from the optical and electrical characteristics of a phase change between amorphous and crystalline phases [1,17]. PCRAM is a candidate for use as the next generation of memory nanodevices, because it can achieve a data-transfer rate greater than gigahertz [18]. The ultrafast switching and reversible phase change of PCMs occurs due to electrical triggering at a nanosecond/femtosecond time scale [12,18].

Chalcogenide (Ch) PCM is an adaptable structure that may allow for the realization of non-volatile and switchable MMs. Ch PCMs are a category of amorphous semiconductors [19] and their alloys, including sulfides, selenides, and tellurides, provide an adaptable structure for tunable MM-based nanodevices. Ch PCMs show nonvolatile electrical/optical switchability, [20] optical nonlinearity, photosensitivity, waveguide optical property, and infrared (IR) transparency, and therefore have applications in memories, solar cells, sensors, bifocal metalenses [21], and photonics [19,20,21].

**Figure 1:**
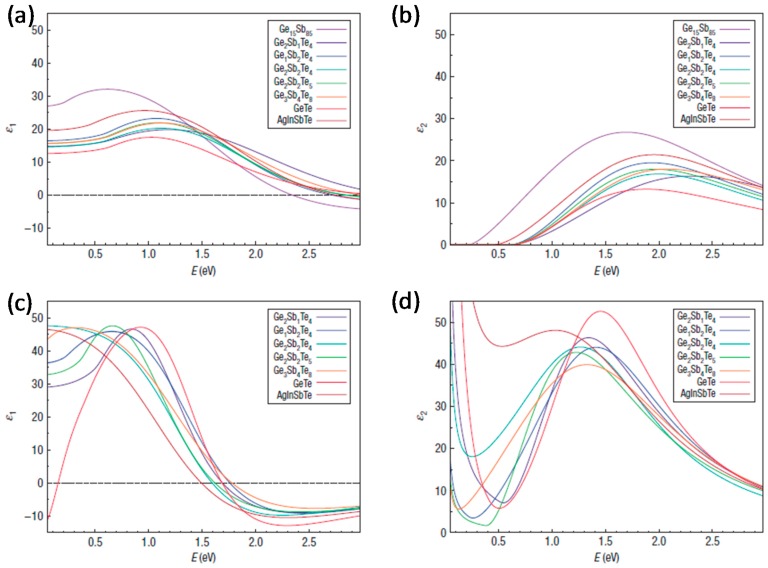
Dielectric function ε_1_(ω) and ε_2_(ω) as optical properties of PCMs. (**a**,**b**) Amorphous PCMs; (**c**,**d**) Crystalline PCMs incorporating Drude-type impact. Adapted from [22], with permission from © 2008 Nature Publishing Group.

The two phases of Ch PCMs differ significantly in their optical and electrical properties. Both the real (ε_1_) and imaginary (ε_2_) parts of their dielectric function vary widely over the energy range from 0.05 to 3 eV (Figure 1). In all Ch PCMs, the dielectric functions of the crystalline and amorphous phases differ widely [22]. Below the bandgap without interband switching excitation, RI of the crystalline phase can increase by 50% because of the resonant bonding [23].

## 2. PCM Material Properties

In PCMs, properties of the crystalline and amorphous phases differ because the crystalline phase undergoes resonant bonding, whereas the amorphous phase undergoes shifts to covalent bonding; the change in bonding modes causes a sharp divergence in optical properties [22,23]. Because of the resonant bonding, crystalline PCMs have higher optical dielectric constants than amorphous PCMs [22]. At low frequencies, crystalline PCMs have free charge carriers, which cause optical absorption, but amorphous PCMs show no optical absorption [23].

Defects in the atomic structure of the Ch PCMs influence their optical properties, such as photosensitivity, photodarkening, photodiffusion, photofluidity, photocrystallization, and birefringence. In Ch PCMs, photosensitivity is the most important property; it is defined as the tendency of a chemical bond to change when it absorbs photons that have a wavelength similar to its bandgap [19]. In a Ch PCM thin film, a tiny laser pulse can cause accretion that enables reproducible neuromorphic shifts that can be used in optical data and image recognition in brain-inspired nanoelectronic devices [24].

Amorphous germanium-antimony-telluride (Ge_x_Sb_y_Te_z_) (GST) is a semiconducting Ch PCM that has been evaluated as a dielectric material. Various compositions of GST have been tested (Table 1); Ge_2_Sb_2_Te_5_ (GST_225_) and Ge_3_Sb_2_Te_6_ (GST_326_) have been extensively used in tunable metasurfaces and rewritable optical data storages [17,25]. In addition to common stoichiometries of GSTs (GST_225_ and GST_326_), Ge_8_Sb_2_Te_11_ (GST_8211_) has been proposed to be utilized as a thin film in nanoantennas [26]. GST can undergo a thermally determined phase transition in response to photothermal heating or Joule heating [27,28]. The transition from the crystalline phase to amorphous phase is caused by a quick increase of temperature to *T*_m_, followed by quenching with a strong and short stimulus; the reverse transition, called annealing, is produced by a smaller stimulus intensity than amorphization [24,29]. Owing to GST’s scalability [30], adaptability with complementary metal oxide semiconductor (CMOS) technology [31], and tunable optical properties [5,29], it has been commercialized for a variety of optoelectrical applications [10]. It has a crystallization temperature of 160 °C and a melting temperature *T*_m_ of 600 °C, so it is used in fast and reproducible electronic memories owing to its thermal stability, switching speed, and rewritability [19].

**Figure 2:**
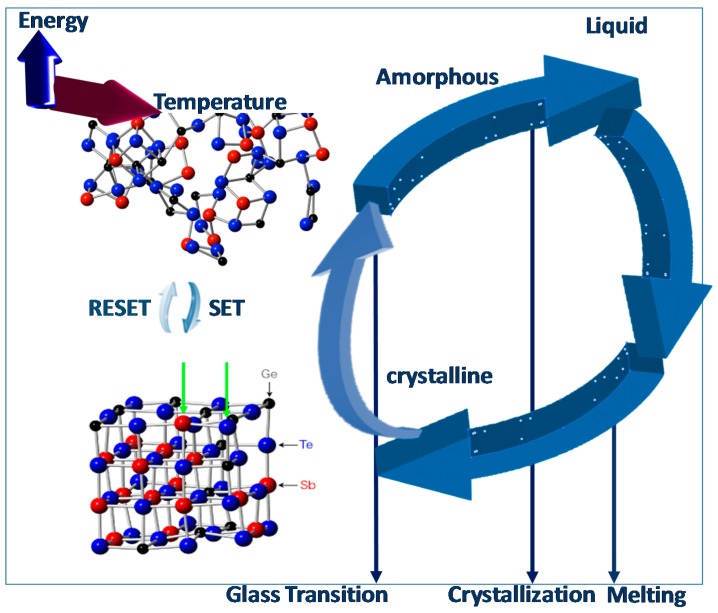
Schematic illustration of phase transition of GST between amorphous and crystalline phases.

GST changes from its as-deposited amorphous phase into a cubic crystalline phase during annealing at a temperature between the glass transition temperature *T*_g_ and *T*_m_, [29] and can be rapidly and reversibly quenched to the amorphous phase by short, high-intensity laser pulses. The two phases differ widely in dielectric properties, and material properties change significantly in both directions (Figure 2). GST with tiny dimensions can be stimulated by light to undergo phase transition. Nanosecond, microsecond, and femtosecond laser pulses stimulate strong switching between the two phases of GST thin film. Multilevel switching is possible by applying femtosecond laser signals [32].

All-dielectric metasurface optical facets have been verified for splitting, filtering, and focusing at wavelengths in the NIR to visible range [33,34]. Active operation has been established by hybridizing a metasurface with a liquid crystal, and by nonlinear optomechanical alignment in the metasurface [35].

## 3. Review

In the following, we review current studies in the field of GST-based active metadevices. The rest of this article considers four major concepts: tunable metasurfaces, tunable absorbers, color-generating tunable metadevices, and other phase-change metasurfaces.

Section 3.1 presents tunable metasurfaces; it begins with a description of active MMs and utiliziation of GST in device applications. Thereafter, we discuss different metasurfaces including grating-based reconfigurable nanostructures and absorber-modulator metadevices. The rest of the section describes various types of metadevices such as Fresnel-zone-plates, dynamic tunable zone-plate devices, planar wavelength multiplexing focusing devices, meta-switches that use split-ring resonators, and nanoantennas.

Section 3.2 reviews tunable absorbers. We first describe GST-based broadband perfect absorber for different wavelength ranges from visible to MIR; the section concludes with a presentation of switchable perfect absorbers and their image capabilities.

Section 3.3 is devoted to color generation. We review GST-based reflective, semi-transparent flexible displays, and GST-based multicolor changeable optical coatings.

Section 3.4 considers other tunable metadevices composed of oxides and Ch PCMs as alternative candidate PCMs with properties similar to those of GST.

### 3.1. Tunable Metasurfaces Using PCMs

Considerable research has been dedicated to switchable metasurfaces [3,4], tunable, and photonic metadevices [2], which are reconfigurable by applying thermal [5,36], or electrostatic signals [37]. By utilizing the reversible and nonvolatile phase transitions of PCM, reconfigurable MMs have been realized to achieve adjustable optoelectrical properties. This section introduces studies of tunable metadevices that use GST as an active dielectric material. Firstly, we review state-of-the-art grating-based all-dielectric metasurfaces [1] composed of a GST layer and the designed MM grating on top of a GST thin film [38]. Thereafter, we focus on various photonic metadevices including lenses, diffractive elements, binary and grayscale devices, optically reconfigurable zone-plate devices and methods to write on them by femtosecond laser pulses on GST layers [29]. Finally, we consider metaswitches [3] of split-ring resonators (SRRs).

#### 3.1.1. GST-Based All-Dielectric Metasurfaces Using Gratings

**Figure 3:**
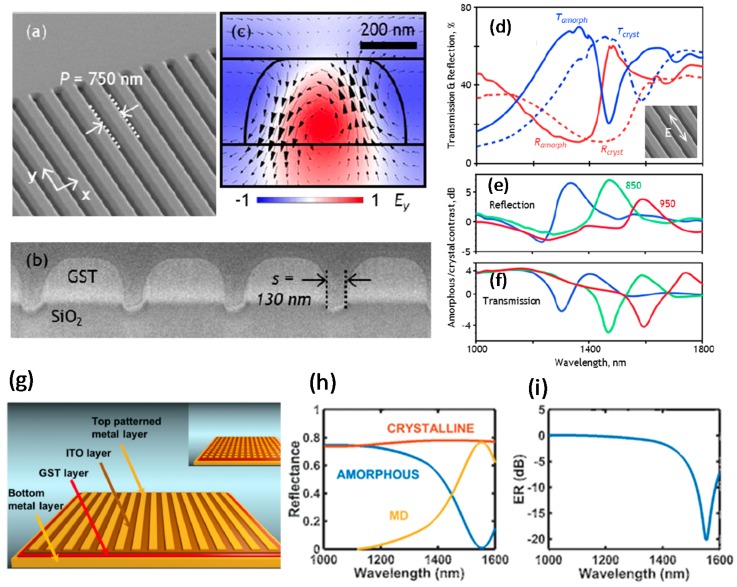
GST-based metasurface with a grating nanostructure. (**a**) Tilted incidence and (**b**) cross-sectional SEM image of the nanograting in a GST_225_ film on an SiO_2_ layer; (**c**) Simulated spreading of y-component of the electric field in the xz surface for a nanograting unit cell at resonance; (**d**) Reflection and transmission spectra of the nanograting based on GST_225_ at both amorphous and crystalline phases. Calculated switching contrast of (**e**) reflection and (**f**) transmission dispersion of the TE mode at both of the GST_225_ phases; (**g**) Schematic illustration of the GST-based absorber-modulator using gratings for near infrared spectra; (**h**) The simulated reflectance curve with strips of Au top electrode and GST_225_ thin film in both recognized phases; (**i**) The optimized extinction ratio (ER) for the maximum modulation depth (MD = 77%) at 1550 nm. (**a**–**f**) are adapted from [1], with permission from © 2016 AIP Publishing LLC; and (**g**–**i**) are adapted from [38], with permission from © 2016 OSA. ER is described by the logarithm of the reflected power ratio, while MD represents the difference between the maximum and minimum reflected power.

Dielectric metasurfaces have been reported with passive, active, and hybrid functionalities. GST_225_ nanograting has been introduced as an all-dielectric metasurface that can be reconfigured by Mie resonance. Near infrared (NIR) transmission and reflection resonances in a dielectric nano-grating metasurface have been reported (Figure 3a,b). GST_225_ thin film was sputtered on a quartz substrate, and a nano-grating metasurface was fabricated by focused ion beam (FIB). These high-quality nanograting arrays of GST-based metasurface showed a non-volatile resonance change. Because transmission and reflectance (T/R) of a transparent layer are related to its thickness and RI, the grating structure demonstrates T/R resonances [39].

The resonance was increased by the nano-grating structure reaching a high-quality factor (Q = λ_r_/∆λ, ≥20), that exceeded the normal amount, where λ_r_ represents the frequency of the resonance and ∆λ is the half maximum width of transverse electric polarized light (TE) at spectra proportional to the grating period *P* (Figure 3a,b) [1].

Numerical simulations (using COMSOL Multiphysics 5.1, COMSOL Inc., Burlington, VT, USA) of electric field distribution in the xz plane (Figure 3c) suggest that the TE resonance is related to the occurrence of anti-phased, vertically dislocated currents adjacent to the nanowires of GST_225_. Therefore, the attained pattern shows the direction of the magnetic field within the nanowires (Figure 3c) [1]. The results show that the resonances of GST-based metasurfaces were optically switched in a nonvolatile manner. Laser excitation at a wavelength of 532 nm caused conversion of the GST_225_ from an amorphous to a crystalline phase. Raster beam scanning leads to an increase in temperature between *T*_g_ and *T*_m_ of GST_225_ nanowire.

The measured TE-mode spectra for the amorphous and crystalline phase of GST-based metasurface showed a considerable modification in T/R at near-resonance wavelengths. This result was obtained due to a change in the complex RI of GST_225_ (Figure 3d) [1]. The spectral dispersion of the TE-mode T/R demonstrated that the grating period (of 850 nm) gave the maximum reflectivity $R_{amorphous}$ of amorphous GST at 1470 nm, but the minimum of reflectivity $R_{crystalline}$ in the crystalline phase; the switching contrast was 5:1, which corresponds to 10·log(*R_amorphous_/R_crystalline_*) = 7 dB (Figure 3e,f). The resonant T/R changes also occurred in the reverse path of the unstructured Ch. The grating-based metasurface revealed high switching contrast, non-volatile, and laser-induced change of the NIR resonant T/R features. In conclusion, this change of RI as a result of a shift in the resonance spectrum caused an alteration in the T/R spectra [1].

#### 3.1.2. GST-Based Absorber-Modulator Using Gratings for NIR

A PCM metadevice has been designed to work as an absorber/modulator in the NIR (1550 nm) [35]. Because optical fibers that work in the NIR spectrum operate at wavelengths between 1530 nm and 1565 nm, the device must be designed to work in this range. The resonator-based absorber/modulator based on GST_225_ thin film was composed of four layers: a metal bottom electrode, a GST_225_ thin film, an indium tin oxide (ITO) film, and a metal top electrode with a patterned structure (Figure 3g) [38]. Reflected light was modulated by exploiting the transition between the crystalline and amorphous phases of the GST_225_ layer. An ITO layer encapsulated the GST_225_ film to protect the active thin film and prevent GST_225_ degradation by exposure to air [10,38]. Plasmonic resonance (oscillating electric dipole) was produced by the incident light on the top pattern of the metadevice [40]. Depending on the phase of the GST_225_ layer, the generated electric dipole coupled to the metallic thin film. In the amorphous phase of GST_225_, an oscillating magnetic dipole perpendicular to the electric dipoles was created by the non-symmetric paired phase of the E-field in the bottom and top layers of the absorber/modulator. However, in the crystalline phase, the induced magnetic dipole decreased significantly [41].

The performance of the absorber/modulator can be measured by considering appropriate figures of merit (FOMs), such as modulation depth [35]

(1) $$MD=\frac{P_{max}-P_{min}}{P_{incident}}=R_{crystalline}-R_{amorphous}$$

and extinction ratio [38]

(2) $$ER=-10\cdot \log\left(R_{crystalline}/R_{amorphous}\right)$$

where $P_{max}$ is maximum reflected power, $P_{min}$ is minimum reflected power, $P_{incident}$ is incident power, and *R_crystalline_* and *R_amorphous_* are modulator reflectance in crystalline and amorphous GST_225_, respectively. High *MD* and *ER* indicate a desirable metadevice; practical applications require *ER* ≤ −7 dB [42].

The reflected, transmitted, and absorbed parts of the energy can be calculated by solving the wave equation of the E-field by using COMSOL Multiphysics with a maximum *MD*.

For both the amorphous and crystalline phases of the GST_225_, the reflectance and *ER* were optimized to have the highest *MD* (~0.77) at 1550 nm (Figure 3h,i). This work demonstrated the feasibility of metadevices based on GST_225_ as absorber/modulators in the NIR spectrum. The metadevice achieved *MD* ≈ 0.77 with *ER* ≈ 20 dB, which is exceptional. Moreover, the designed metadevice had good potential to be switched by an electrical stimulus, as do PCRAMs [38].

#### 3.1.3. GST-Based Reconfigurable Metasurfaces and Metadevices

**Figure 4:**
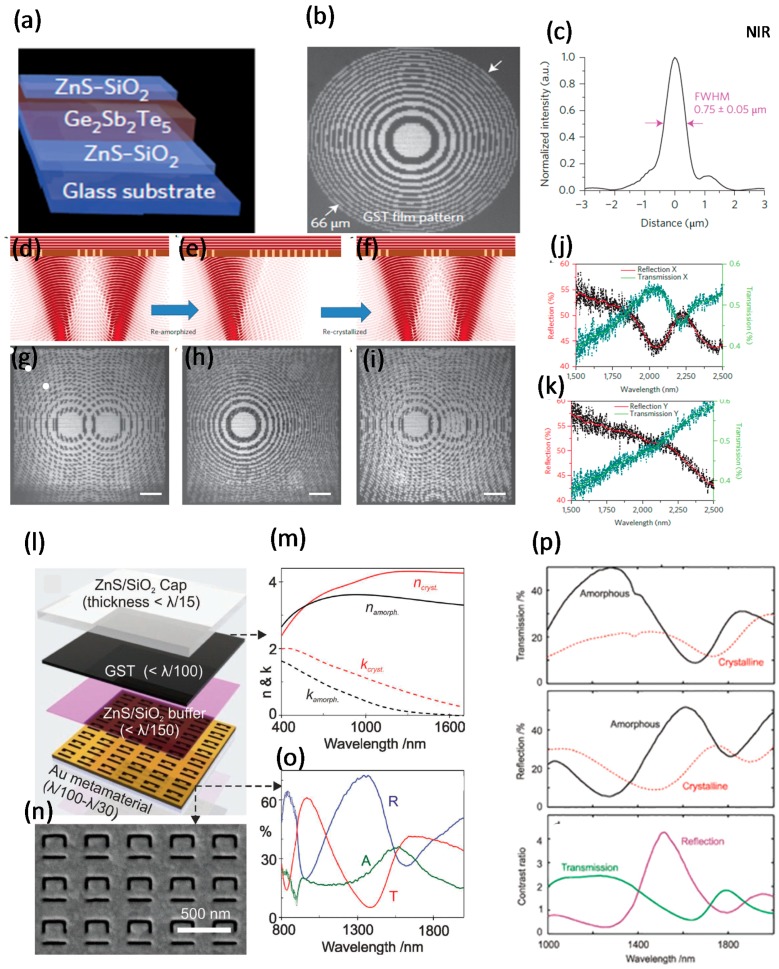
GST-based various photonic meta-devices. (**a**) Schematic demonstration of the metadevice structure; (**b**) Fresnel-zone-plate pattern, which was written in the GST layer of binary and grayscale metadevice; (**c**) The cross-sectional strength of optical hotspots that were concentrated by Fresnel-zone-plate; (**d**–**f**) Tunable and active zone-plate metadevice; two overlapped Fresnel zone patterns are (**d**) written, (**e**) erased, and (**f**) rewritten; (**g**–**i**) Overlapped Fresnel zone patterns figured during three steps of writing, erasing, and restoring corresponding to (**d**–**f**); (**j**,**k**) R/T spectra of the written dielectric material into the GST layer for (**j**) horizontally and (**k**) vertically polarized light; (**l**) Multilayer split-ring resonator of GST-based meta-switch; (**m**) The RI and k of GST under both amorphous and crystalline phases measured by an ellipsometer; (**n**) SEM image of the fabricated metadevice; (**o**) Reflection, transmission, and absorption curves of the meta-switch with vertically polarizing light to the splits of the ring resonator, before using a buffer layer, GST film, and the protective layer; (**p**) Optical transmission, reflection, and their modulation contrast related to GST phase shift in both phases of GST layer of the hybrid metadevice. (**a**–**k**) are adapted from [29],with permission from © 2017 Nature Publishing Group; and (**l**–**p**) are adapted from [3], with permission from © 2013 WILEY-VCH Verlag GmbH & Co. KGaA, Weinheim.

Active photonic elements have been achieved by printing a tunable dielectric PCM [29]. Different reconfigurable photonic devices using GST_225_ layers have been presented, including binary and grayscale devices such as Fresnel-zone-plates, dynamic tunable zone-plate devices, planar wavelength multiplexing focusing devices, and writable dielectric MMs. The possibility of nonvolatile optical properties with diffraction-limited resolution (DLR) was demonstrated [29]. When the GST_225_ changes from amorphous to crystalline, the complex RI changes dramatically; based on this phenomenon, bright marks were constructed in reflection images in an apparatus composed of a DLR optical pattern generator and a femtosecond laser. A 70 nm GST_225_ film was packed between ridges of a ZnS-SiO_2_ film deposited on a glass substrate (Figure 4a). A planar dielectric MM with optical resonances in NIR was optically written, erased, and rewritten [29].

Binary and grayscale photonic devices have been produced using a GST_225_ thin film [29]; a Fresnel zone was engineered to assign each equivalent intensity to a focal point. An optical image of Fresnel-zone-plate contains a set of consecutively impenetrable and transparent rings expanded to focus light by diffraction (Figure 4b). The hot spot had an FWHM of ~0.75 ± 0.05 µm (Figure 4c). They also confirmed that chromatically selective and chromatically rectified lenses can be written to produce planar wavelength multiplexing devices [29].

A dynamic and reconfigurable zone-plate device was created from a GST_225_ layer with enormous number of switching cycles between two different phases [29]. A write-erase-rewrite reconfiguration cycle with a double Fresnel-zone-plate pattern was achievable by changing the phase of the GST_225_ (Figure 4d–f). First, two superimposed Fresnel zone patterns with different foci were fabricated on a plane (Figure 4d), one of the patterns was then removed by returning it to the amorphous phase (Figure 4e), and the pattern was then rewritten by recrystallizing it (Figure 4f). The superimposed Fresnel zone pattern were imaged under first-time writing (Figure 4g), after erasing the second pattern (Figure 4h), and after restoring it (Figure 4i) [29].

The large disparity in complex RI in the NIR between the amorphous and crystallized phases is the most important feature of GST_225_. Using this key feature, dielectric MM arrays with resonance can be written. A dipolar dielectric MM composed of an array of crystalline attachments in the amorphous phase was also demonstrated. The dielectric MM with an embedded pattern in the GST_225_ consists of two signs of phase shifts, one horizontal and one vertical (Figure 4j,k). Amounts of T/R calculated using a Fourier-transform infrared (FTIR) displayed the resonant T point and R slope for light polarized along the long axis [29].

To conclude, a reported active tunable optical device that can be controlled by light include rewritable devices, holograms, and a resonant MM. Nonvolatile switchable light modulators, signal distributors, tunable elements, and reconfigurable NIR photonics are some plausible applications of this technology [29].

#### 3.1.4. GST-Based All-Optical Meta-Switch Using Split-Ring Slots

The resonant optical properties of plasmonic MMs are governed by the near-field dielectric environment. Therefore, any alteration of the RI or absorption coefficient of a neighboring nanolayer generates gigantic variations in the T/R characteristics of a hybrid assembly [3]. Active plasmonic MMs show volatile responses to external stimuli such as heat, light, current, voltage, or electric and magnetic fields [2,4,5,6,36,43,44]. To develop a nonvolatile response, an all-optical switch using GST_225_ with a nanostructured plasmonic MM that generates extreme and flexible switching within NIR and MIR wavelengths has been reported; an optically stimulated phase change in GST_225_ media specifies a platform for nonvolatile MM [3]. The optical Ch-based metamaterial modulators showed a controlled hysteretic response because of insulator-metal transitions phases with a rapid, vigorous, consistent, and non-volatile phase shift. In a hybrid Ch configuration of a metadevice, the GST_225_ underwent a nonvolatile switch from NIR to MIR wavelengths under stimulation by a nanosecond pulse. A GST_225_ thin film was sandwiched between two buffer and capping layers of ZnS/SiO_2_ (Figure 4l) [3]. The extinction coefficient *k* and the strength η of light absorption at a given wavelength of the GST_225_ layer were measured in both phases (Figure 4m). SEM images confirmed that FIB milling could fabricate split-ring resonators (SRRs) well on a multilayer hybrid structure (Figure 4n). Feeble coupling of the excitation mode to free-space radiation modes inside SRR asymmetric arrays caused restricted spectra of *R*, *T*, and absorption *A*, and Fano-type dispersion in an NIR-resonant MM (Figure 4o) [3]. The laser-induced spectral dispersion of the metadevice demonstrated a huge change in *R* and *T* in both phases of the GST_225_ layer; as a result, the SSRs underwent a huge modulation contrast related to GST_225_ phase shift (Figure 4p) [3]. The absolute *R* and *T* of SRRs made of GST_225_ at definite wavelength bands varied by about a factor of four, and optimization of the designs of SRRs increased the distinction [3]. The transition was induced optically in both the amorphous and crystalline phases [45].

#### 3.1.5. GST-Based Rectangular Nanoantenna

Absence of losses is one of the main requirements for realization of active MMs with specific designs [23]. Plasmonic antennas made of metallic nanostructures have been developed to exploit their localized field increase by light [12]. Their resonance frequency can be controlled by varying their thickness or by changing the dielectric environment [4,12,46,47]. The field confined in the metal surface of plasmonic metallic nanoantennas can be strengthened by selecting the geometry, material, and substrate, but their resonant wavelength is fixed [48]. Optical properties can be tuned by using MMs and active plasmonics. Using PCMs allows nonvolatile and reversible control of the optical reaction to the nanoantennas. Among all GST-based PCMs, GST_326_ incurs the smallest loss in the MIR. The real part of the RI of GST_326_ varies widely over MIR wavelengths, but its imaginary part is negligible. The two phases of GST_326_ film have different real components of RI, and this difference can be exploited to control the resonance frequency in nanoantennas, and, because the imaginary part of the RI is small, resonance damping in the MIR spectrum is prevented.

**Figure 5:**
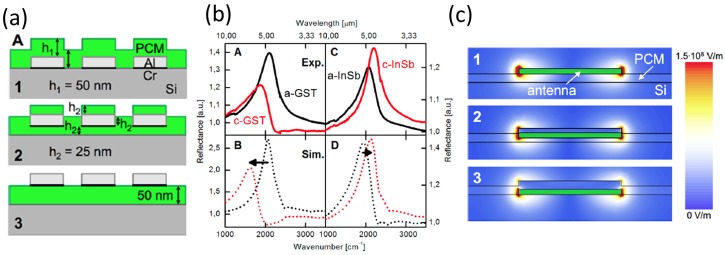
GST-based resonance antenna. (**a**) Schematic graph of the designed nanoantennas below, amongst, and on top of the GST layer; (**b**) Measured and simulated reflectance spectra for the amorphous and crystalline GST layer; (**c**) The first resonant field distribution for various types of the designs below, inside and on top of the GST film. Adapted from [12], with permission from © 2013 American Chemical Society.

The plasmonic nanoantenna arrays have been designed below, inside, and on top of the GST_326_ layer as a PCM material to regulate the amount of the resonance to the upper and lower limits of the wavenumbers (Figure 5a). The antenna was designed to function in the MIR to prevent damping of the resonance signal and to reduce absorption loss to an insignificant level [12]. In the MIR, the real part of the RI is larger than it is in the visible range, and lower than the optical bandgap, so the imaginary part of ε is small [22]. By changing the RI of the covering layer of a nanoantenna, the resonance wavelength in the MIR can be increased; as a result, the FOM varies. Here, FOM is defined as the ratio of the resonance range to the full width at half maximum of the resonance peaks (FWHM) [12]:

(3) $$\mathrm{FOM}=\frac{\mathrm{Switching}\;\mathrm{range}\;\mathrm{of}\;\Delta v\;\left[cm^{-1}\right]}{FWHM\;\left[cm^{-1}\right]}.$$

The dimensions of the antennas and the lattice periods were engineered to achieve spectrally narrow resonance by exploiting Fano interference between the lattice and antenna resonances [12]. The GST_326_ thin film was deposited on Si substrates, and Al was selected as the nanorod material to reduce metal diffusion into the PCM (compared to Au). The phase change was achieved by annealing the fabricated structure at 160–180 °C. Crystallization of the GST_326_ layer during heating changed the covalent bonding of the amorphous phase to resonant bonding [19,49]. FTIR analysis demonstrated a parallel polarization of the incident light through the long axis of the antennas (Figure 5b, Part A) [12]. The resonance shifting of nanoantenna was represented by reflectance spectra for both phases of GST_326_ in an antenna with a length of 600 nm; the change of the GST_326_ from amorphous to crystalline caused a red shift in the resonance frequency (Figure 5b, Parts A,B). The geometry of the sample matched the design in Figure 5a. Spectra were simulated using finite difference time domain method (FDTD) before and after thermal annealing (Figure 5b, Part B) [12]; the calculated absolute values of resonance frequency differed from the measured values for several reasons, including the simplified simulation model, neglect of the adhesion layer, and presence of native oxides. Moreover, the spectra from FTIR measurement broadened as a result of the effects of sample deficiencies and inhomogeneities [12]. FOM was increased by decreasing the lengths of nanoantennas and by changing their positions. The calculated FOMs for nanorod designs below, inside, and on top of the GST_326_ were 0.47, 0.87, and 1.03, respectively. The highest tuned FOM was 1.03 for nanorods on top of the GST_326_ layer with a maximum resonance shift of 19.3% [12].

Absolute values of the dispersed electric field of the first resonant mode were simulated by numerical analysis of the nanoantenna array, with antennas under, within, and on top of the GST_326_ layer. The antennas arranged on the top showed the most powerful overlap of the scattered field and the GST_326_ layer (Figure 5c). These studies have achieved nonvolatile and adjustable loss-less resonance swapping of plasmonic nanoantenna in the MIR domain. This swapping was obtained by using the GST_326_ thin film to control the RI of the nanorods. Electromagnetic (EM) waves were damped only slightly because the imaginary part of the dielectric was tiny [12].

### 3.2. Tunable Absorbers Using PCMs

MMs with various resonances can be used to create a broadband absorber [50,51], so these metadevices have been developed; each operates in a specific wavelength [52,53]. Recently developed MM-based perfect absorbers (MPAs) have polarization independence, high light absorbency, exotic properties, and unity absorptivity of electromagnetic (EM) waves [28]. EM wave absorbers are classified as either resonant absorbers or broadband absorbers.

Absorption is detrimental. It occurs with a cavity thickness much smaller than the wavelength, because of the immediate shift in phase of the incident light by the metasurface [54]. Solar cells, RI sensors, and detectors are realized by using substances that absorb EM in the IR and visible ranges of the spectrum. Introducing GST_225_ to the plasmonic resonator structure of an MM leads to an active metadevice such as a tunable absorber or switched modulator by modified optical/electrical characteristics [38]. Tunable absorbers have potential applications as thermal emitters, electro-optic switches [46], and adjustable sensors [55].

#### 3.2.1. GST-Based Broadband Perfect Absorber over Visible Range

Here, we discuss recent work on GST-based efficient MPAs with reconfigurable characteristics. The major parameters that must be considered when designing MM absorbers are their polarization-dependency, incidence angle, and bandwidth. Perfect absorbers are the most desired type; they have been studied at microwave, terahertz, and IR frequencies [28,56]. Correspondingly, MPAs that have dual-band [57], triple-band [58], and tunable-band [59] characteristics at terahertz frequencies have been fabricated. However, the development of an MPA with broadband absorbance in the visible range is challenging [28].

The insulator layer of an MM absorber with a metal-insulator-metal (MIM) structure is a material with high imaginary part of RI, and is sandwiched between two metals. The top metal is a patterned layer and the bottom metal is an unpatterned thin film that works as a mirror to eliminate *T*. The electric and magnetic dipolar resonances are produced by the patterned metallic layer and dielectric constant of the insulator film, respectively [28]. A dielectric layer with high imaginary part of RI was introduced to increase *A* by connecting to a cavity resonance [22].

**Figure 6:**
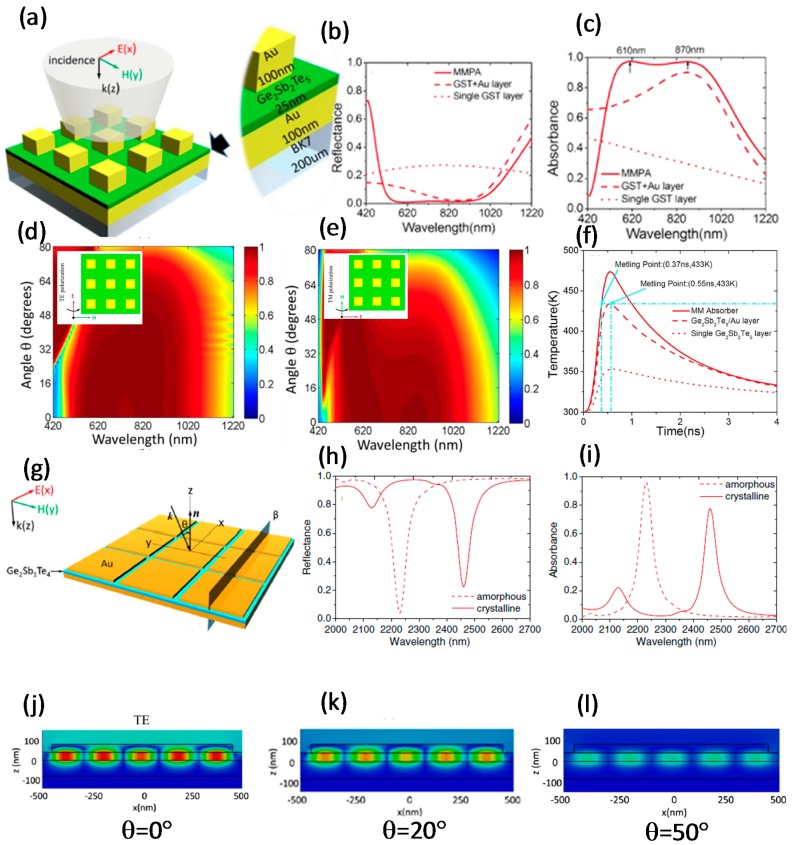
GST-based tunable absorbers. (**a**) Structural representation of the broadband absorber with incident light polarization; Simulated spectra of (**b**) reflectance and (**c**) absorbance for three configurations including a perfect absorber, hybrid, and single GST layer; Simulated absorbance peak distribution for (**d**) TE and (**e**) TM polarization of GST amorphous film; (**f**) The simulated temperature of one pulse through the perfect absorber, hybrid, and single GST layer; (**g**) Graphical representation of the absorber structure with the incident light polarization while the incident angle of the plane wave on the surface is θ; Simulated (**h**) reflectance and (**i**) absorbance spectra of GST layer at perpendicular incidence; Scattered magnetic field of TE polarization at different angles of (**j**) θ = 0°, (**k**) θ = 20°, (**l**) θ = 50°. (**a**–**f**) are adapted from [28], with permission from © 2014 Nature Publishing Group; and (**g**–**l**) are adapted from [59], with permission from © 2013 OSA.

A dynamic MPA with active tunability has been achieved by using PCM as a dielectric layer. In an MPA with arrays of Au squares on an Au/GST_225_ layer, each unit cell of the arrayed squares works as an optical resonator (Figure 6a) [28]. 3D-FEM simulations were used to optimize the absorber for perpendicularly incident TE plane waves. The spectra of reflectance and absorbance for the metadevice, hybrid, and single GST_225_ layer were simulated at normal incidence (Figure 6b,c). The reflectance of the MPA was extremely low over the entire visible spectrum due to impedance matching to vacuum (Figure 6b). Consequently, the absorbance was maximal over the entire visible range (Figure 6c). Near-perfect absorption of 96.8% and 96.2% were obtained at wavelengths of 610 nm and 870 nm, respectively (Figure 6b,c). Compared to other structures, the reflectance and the absorbance of a single GST_225_ film were at their maximum and minimum respectively, due to the lack of metallic layer (Figure 6b,c) [28].

The absorbance of an MPA is independent of the incidence angle and polarization the light [60]; therefore, to confirm that the device is an MPA, the angular dispersion of the absorbance peaks at both of the TE and transverse magnetic (TM) polarizations were simulated. In the simulations, the absorbance of an amorphous GST_225_ thin film at incidence angles 0–80° remained stable all over the visible and NIR regions (Figure 6d,e) [28]. Because the imaginary part of the dielectric permittivity for GST_225_ is extremely high in the visible regime, absorbance was high at incidence angles >80° for both electric and magnetic polarizations [28].

In the visible range, GST_225_ has a large imaginary part of RI, so transition between the two phases is rapid, and the wideband absorbance increases. Simulation results suggested that the designed metadevice satisfied the basic requirements of a broadband MPA over a wide range of incidence angles in the visible and NIR spectra [28].

To represent the rapid phase transition of three different designs of the GST_225_ layer (single GST_225_, MPA, and GST_225_/Au layer) from amorphous to crystalline, the temporal deviation of temperature was simulated using FEM in COMSOL (Figure 6f). GST_225_ increases the absorption of EM radiation in the MPA by fast heating and therefore rapid phase shift [28]. The MPA absorbed in the metallic parts by converting the absorbed energy to heat [60]. Simulation results suggest that the temperature of the amorphous dielectric layer is related directly to the radiation flux.

During one pulse, the different structures of amorphous GST_225_ reacted differently (Figure 6f). The temperature of GST_225_ in the MPA shows that, due to heat radiation, the MPA cooled before the next pulse arrived (Figure 6f) [28]. A big difference between the temperature of the absorber and the single GST_225_ thin film confirmed that the MPA increased the heat flux through the amorphous GST_225_ layer. Overall, the thermal phase change of the MPA worked as an ultrafast photo-thermal reconfigurable nanophotonic with several merits. GST-based perfect absorbers can be used as switches that consume ultralow power and that do not need a permanent source of energy to sustain the phase [28].

#### 3.2.2. GST-Based Tunable Perfect Absorber over MIR Range

Because a specific metal layer MM combined with GST can acquire an extensively flexible Fano resonance, GST can be used as a tunable absorber [46]. The metadevice continues to absorb efficiently over a wide range of incident light angles for TE and TM polarizations. The designed structure for a tunable perfect absorber is similar to the broadband perfect absorber in the visible range [28]. The absorber structure is a combination of gold square arrays separated from a continuous Au thin film by a GST_214_ layer. Switching the GST_214_ between its two phases changed the absorbance peak changed by 10%. The absorbance showed a significant overlap between TE and TM polarizations across a wide range of incidence angles. The metadevice can be schematically illustrated by the incident light conformation (Figure 6g) [59]. The simulated reflectance (Figure 6h) and absorbance (Figure 6i) for the GST-based absorber throughout the MIR domain under normally incident light were obtained using 3D-FDTD simulation. The wavelength of the peak of the strong absorbance in the MIR range was increased by switching the GST_214_ layer from its amorphous to crystalline phase (Figure 4i) [59]. Entire spreading of the magnetic field for TE polarization with angles of 0°, 20°, and 50° was simulated using FDTD to examine the absorbance and intensity of the magnetic resonance (Figure 6j–l) [59]. The absorber retained localized magnetic field strength. Accordingly, the orientation of the magnetic resonant dipole was maintained when incidence angle was large. Complete E and H intensity distributions show that the EM field can be competently confined inside the absorber by various incidence angles [59].

#### 3.2.3. GST-Based Switchable Perfect Absorber with Image Capability

**Figure 7:**
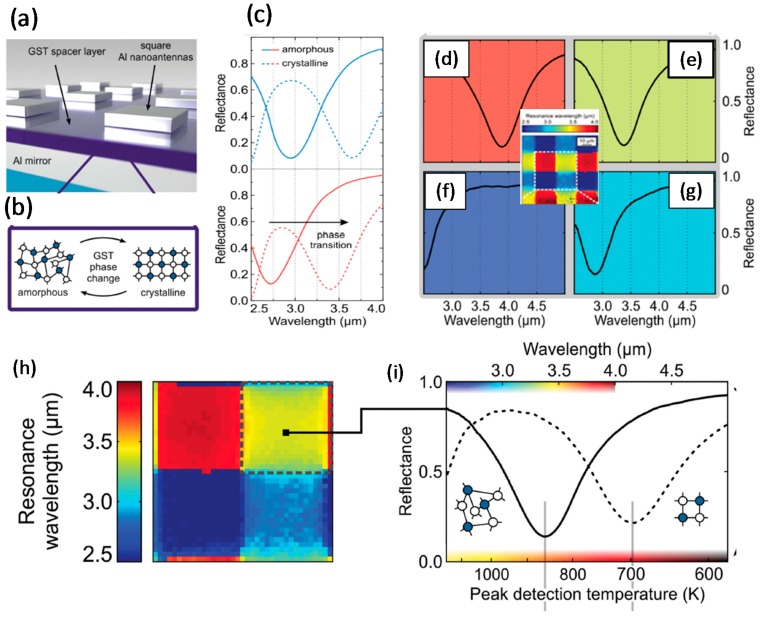

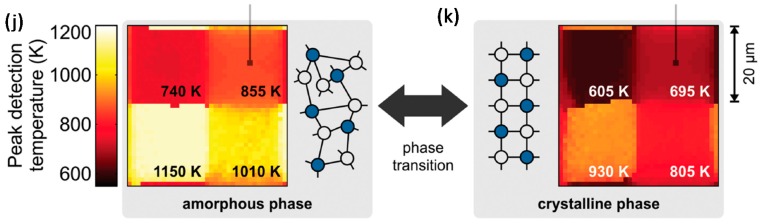
GST-based perfect absorber with image capability. (**a**) Illustration of a switchable absorber; GST is packed between Al nanopatterns and Al mirror; (**b**) Sketch of GST phase change between two amorphous and crystalline phases; (**c**) Reflectance curve in MIR spectral region corresponding to the GST layer in both amorphous and crystalline phases; the higher resonance wavelengths are produced by the larger dimension of antennas; (**d**–**g**) Typical reflectance spectra of the four specific pixels illustrated in the inset, showing the imaging capability of the perfect absorber; (**h**) The resonance wavelength of four separate MIR absorption ranges; (**i**) Typical reflectance spectra of the yellow color-coded resonance wavelength for GST in both of the amorphous and crystalline phases. Peak detection temperature for the (**j**) amorphous and (**k**) crystalline phass of the GST layer. Adapted from [64], with permission from © 2015 John Wiley and Sons.

A switchable MIR plasmonic perfect absorber (PPA) with temperature selectivity and thermal imaging ability has been obtained by using PCM as a switchable material between two Al layers. The representative stoichiometry (GST_326_) of the Ch-PCM achieved lower loss than GST_225_ in dynamic plasmonic metadevices [61,62,63]. GST_326_ as a spacer layer was sandwiched between two Al layers, one as a mirror and one as a nanoantenna array (Figure 7a). The absorber pixel was determined by the length of Al nanopatches to define the resonance wavelength, and the dimensions of the arrays to define pixel size. GST_326_ served as a dielectric layer to obtain switchable and band-selective operation of the PPA by phase transition between amorphous and crystalline phases (Figure 7b). The use of GST_326_ achieved strong divergence of reflectance at resonance and noticeable spectral shifts of up to 25% [64].

Multispectral imaging has been realized by using pixels with spectrally diverse absorption bands on pixels <10 µm in size [65]. Al was used as the material for nanoantennas to make it CMOS-compatible. The geometry of the PPA displays perfect absorption characteristics in MIR range (a wavelength of ~3–5 μm) [64]. Under heat treatment (300–700 °C), an (or any) object shows thermal emission peaks within the MIR spectral range. Each pixel in the PPA absorbed radiation at specific resonance wavelength that was related to the temperature of the pixel; therefore, the device could be used as a selective thermal imaging metadevice (STIM). The measured and calculated reflectance of the STIM with geometrical parameters of the nanoantennas revealed a small reflectance that corresponded to high resonant absorption in the MIR (Figure 7c). Changing the phase of GST_326_ caused a 25% red shift in the resonance wavelength of the PPA, with high absorbance (Figure 7c, dashed lines). Consequently, each length of nanoantenna could switch between two MIR absorption bands [64].

Multiple imaging capability (MIC) is an additional feature of the PPA that confines numerous wavelengths of a radiation on a specific device. MIC capability can be realized in a PPA by using various nanoantenna arrays with different side lengths. Four distinct pixels offered >80% maximum absorbance at resonance and supported a 20 μm pixel size (Figure 7d–g) [64].

To determine whether the PPA could be used for thermal imaging (TI), Planck’s law was used to calculate the resonance wavelength with four MIR absorption bands (Figure 7h). Thermal radiation of a heated specimen with the required temperature was extracted effectively for each resonance wavelength. A typical reflectance curve of a distinct pixel (Figure 7h) for both phases of GST_326_ has been illustrated (Figure 7i). GST_326_ had TI ability in the amorphous phase at 740–1150 K and in the crystalline phase at 605–805 K (Figure 7j,k).

In the amorphous phase, the highest heat generation occurred due to absorption of the highest incident energy by the closest pixel to the peak of the thermal radiation (Figure 7j). However, in the crystalline phase, compared with the amorphous phase, the TI ability was demonstrated within different temperature ranges, but with a reasonable efficiency of 75% (Figure 7k). This device was a switchable PPA in the MIR range with multispectral TI ability, temperature selectivity, switchability, high efficiency, and polarization independence achieved in 10 μm absorber pixels.

### 3.3. Tunable Colors Using PCMs

Resolution and efficiency of low-dimensional color filters are being improved. Metallic nanostructures have been used to increase plasmonic color generation using surface plasmons. Plasmonic nanostructures including nanoantennas, metallic gratings, nanohole arrays, and nanodisks lead to high T/R efficiency, and electro-optic changes [66].

Active materials such as PCMs with plasmonic effect and volatile phase shifts have been manipulated to achieve color-change systems with applications in display technology [67,68]. PCMs show vigorous optical modulations including noteworthy resolution with low power consumption [69]. In PCMs and specifically GST_225_, color switching can be exploited by the tremendous difference between real and imaginary parts of RI in two optical phases [70]. The calculated XY color gamut of GST_225_ on a chromaticity diagram with different thicknesses has shown a wide range, which indicates good color modulation [71].

#### 3.3.1. GST-Based Reflective and Semi-Transparent Flexible Display

A GST_225_ thin film has been used to develop an optoelectronic reflective display that has a wide range of applications in display technologies. The device had good potential to achieve high resolution, fast switching, and low power consumption [10].

**Figure 8:**
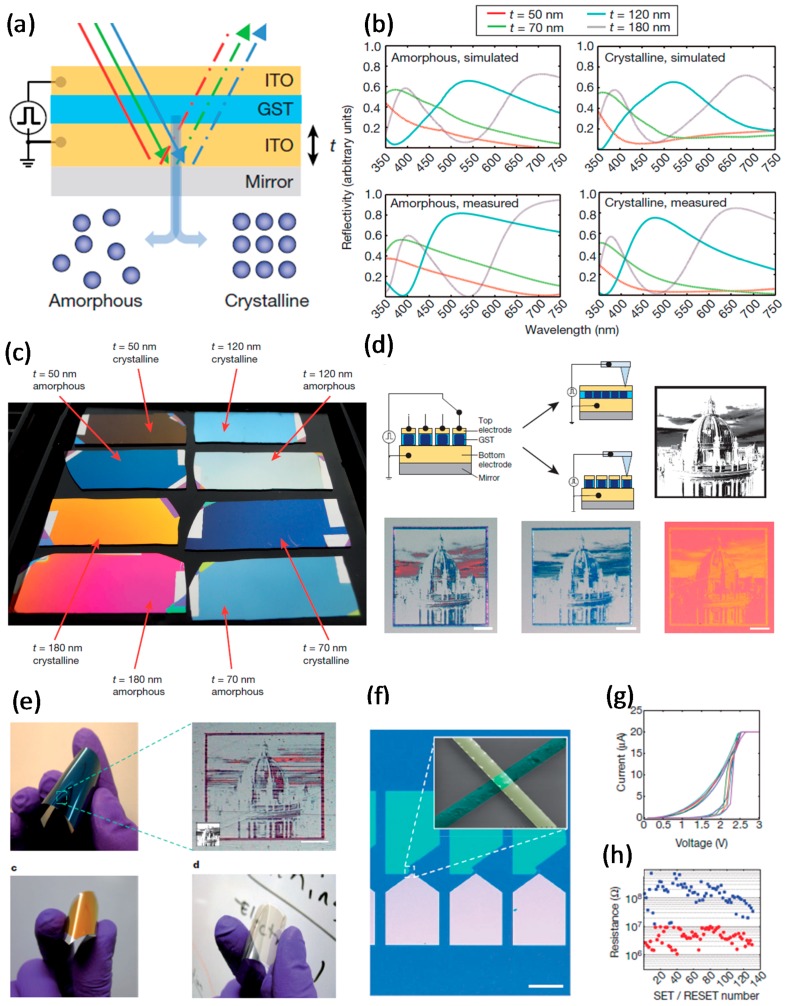
Color generation tunable metadevices. (**a**) Representation of a thin film pack with GST layer sandwiched between two ITO films; (**b**) Reflectivity spectra for amorphous and crystalline phases of GST with simulation (top part) and measurement (bottom part); (**c**) An illustrative example of four various films with Pt/ITO/GST/ITO structure and different thickness of ITO (**t**), which defines the reflective color; (**d**) Electrical alteration of the color and reconstruction of the patterned image using CAFM; (**e**) The flexibility of the reflective display on a plastic substrate. (**f**) An optical image of the crossbar device; (**g**) *I*–*V* curve of the electrically switchable device; (**h**) More than 100 set/reset cycles of the fabricated crossbar device. Adapted from [10], with permission from © 2014 Nature Publishing Group.

The GST_225_ thin film was packed between two conductive ITO layers on a Pt-coated Si wafer (Figure 8a). The color was changed via switching the GST_225_ phase from amorphous to crystalline. Phase modulation to change the RI of the GST_225_ layer also affected the reflective spectrum of the display device (Figure 8b) [10], and the thickness dependence of the color change was demonstrated (Figure 8c) [10].

An optical computational model based on a transfer matrix was used to calculate T/R and transmission coefficients of the electromagnetic field under visible incident light [10,72]. The effect of GST_225_ thickness was examined and optimized together with the dielectric layer. Reducing the thickness of GST_225_ increased the contrast and decreased the electrical switching power. Electrical switchability was achieved using a pixelation procedure with conductive atomic force microscopy (CAFM) (Figure 8d) [10].

Electrically triggered color change in the GST-based display was demonstrated by displaying a patterned image on the GST_225_ stacked device. To achieve transmissive color change, the layer was printed on a transparent substrate and the pixelated image was achieved using CAFM in a similar way to the reflectance image. The flexibility of the semi-transparent reflective display was appraised using a plastic substrate and very thin GST_225_. An ultrathin flexible device with good wide-angle color uniformity (Figure 8e) was developed; it has possible applications in touchable displays [10].

Crossbar devices with ITO/GST_225_/ITO configuration were fabricated with 300 nm × 300 nm active areas to demonstrate optoelectronic functionalities (Figure 8f). The *I*–*V* curves for positive sweeps and data endurance properties had PCRAM properties (Figure 8g,h). An optoelectronic active device was achieved with low energy consumption and a low-dimensional GST_225_ film. The extremely thin layer of GST_225_ showed high optical contrast between two amorphous and crystalline phases. The image rendered on a flexible device was comparable to that on a rigid device [10].

#### 3.3.2. GST-Based Multicolor Changeable Optical Coating

**Figure 9:**
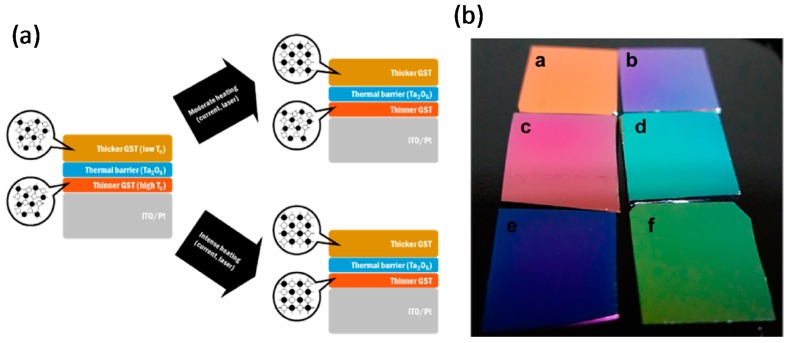

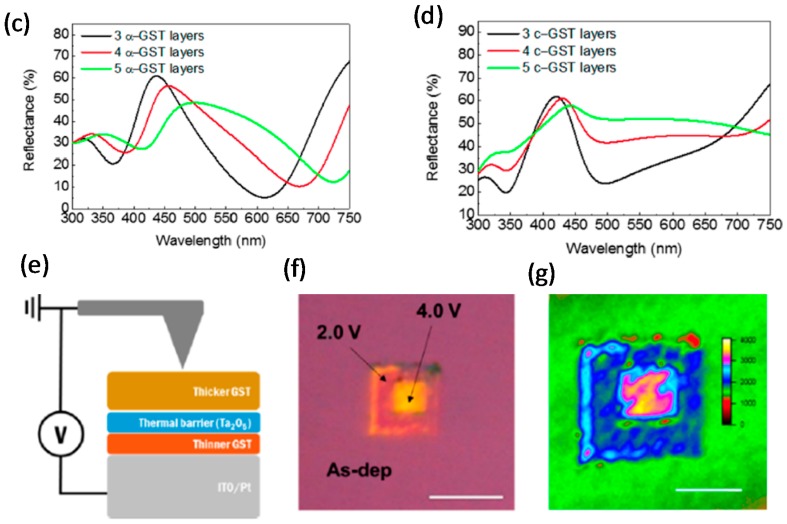
GST-based multicolor changeable optical coating. (**a**) A depicted working mechanism of two GST layers separated by an oxide under amorphous and crystalline phases; (**b**) Three different groups of GST/oxide/GST samples under the crystalline, semicrystalline, and amorphous phases. The simulated reflectance of multilayer coatings under (**c**) amorphous and (**d**) crystalline phases; (**e**) Sketch of CAFM for the electrical response of the device. Three distinct color phases of the samples obtained using (**f**) an optical microscope and (**g**) a confocal laser scanning microscope (CLSM). Adapted from [73], with permission from © 2016 American Chemical Society.

A multilayered optical coating composed of an optical cavity of Fabry-Perot (FP) type and several layers of GST_225_ film has been reported; the color was controlled by the FP interface, and the GST_225_ thin film caused a strong interference effect and therefore operated as an absorbing dielectric [70]. The colors produced were very diverse, possibly as a consequence of optical attenuation and interface phase changes of the GST_225_ layer [73]. FP interference and a the high absorbance of the dielectric layer may be the main reasons for the color change. Diverse colors were achieved using different thicknesses of the transparent spacer layer (ITO) and a bi-stable GST_225_ film. Several phases could be achieved by controlling external triggers such as thermal stimulus, electrical current, and optical laser intensity (Figure 9a) [74]. Samples with different colors were achieved by adjusting the ITO thickness and the annealing temperature, as shown in Figure 9b. The three samples in the first column (Figure 9a,c,e) and the three samples in the second column (Figure 9b,d,f) are representative of stacks with 150 nm and 200 nm thicknesses of the ITO layer, respectively. Additionally, the two samples located in the first row (Figure 9a,b), second row (Figure 9c,d), and third row (Figure 9e,f) depict the crystalline, semi-crystalline, and amorphous samples, respectively (Figure 9b). The phase transition of each layer in the multiple stacks causes the generation of different colors (Figure 9b), so changing the temperature caused the reflectance curve of the GST_225_ layer to undergo a huge shift

(4) $$∆R=(R_{crystalline}-R_{amorphous})/(R_{crystalline}+R_{amorphous})$$

due to its phase transition [73].

The measured and simulated ∆*R* were in good agreement for various types of annealed samples. As the number of GST_225_ stacks increases, the disparity of the total reflection increases due to changed colors [10,70,73]; this relationship should be optimized.

As the number of deposited amorphous GST_225_ films was increased, the total thickness increased, so the reflectance decreased (Figure 9c), but the curve shifted to higher wavelengths; i.e., the achieved colors were deviated from the defined colors. The reflectance of amorphous GST_225_ with the same number of layers changed when it changed to crystalline. Increasing the number of layers of crystalline GST_225_ film weakened the absorption peak at wavelengths from 350 and 500 nm, so the reflection was a metallic silver without any contrast (Figure 9d) [73].

The proposed structure has possible uses as an optoelectronic display, optical storage device, or multilevel recording to increase data density. To appraise these possibilities, CAFM was used to analyze the multiphase optical recording. Bias was applied to the bottom electrode while the tip was grounded (Figure 9e) [73]. A bias was applied to the crystalline region to induce Joule heating to change the phase. Optical (Figure 9f) and confocal laser scanning (Figure 9g) images detected a distinct reflectance change under change in the bias [73]. These results demonstrated that several colors can be produced using multilayered coatings of GST_225_ film, then triggering them by annealing or by electrical current.

### 3.4. Other Phase-Change Metasurfaces

There is a debate regarding oxide-based metadevices. Vanadium oxide (VO_2_) has been recently classified as PCMs owing to its temperature-induced optical properties. In contrast, because it does not satisfy the basic requirements of PCMs for active metadevices such as nonvolatile and reversible response to stimuli, it is a controversial PCM. VO_2_ switches from an insulating phase to a metallic phase when heated, so its RI changes greatly. However, the phase transition is volatile in VO_2_, but non-volatile in Chs [75]. Because this phase change is volatile, VO_2_ is not categorized as a real PCM, but is the most widely used material in active optical metasurfaces [76]. Its reversible phase shift on heating is from a monoclinic insulator to a half-filled metallic rutile phase; as a result, its resistivity decreases by more than five orders of magnitude [27]. The phase transition of VO_2_ provides access to a wide range of complex RIs and is therefore applicable to reconfigurable optical devices.

**Figure 10:**
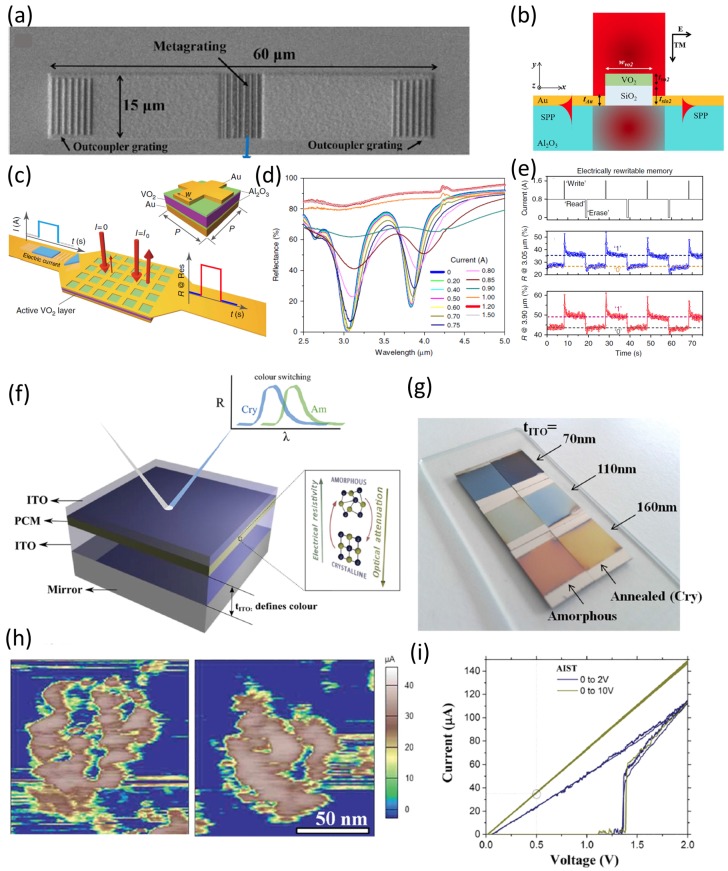
Other phase change metasurfaces. (**a**–**e**) VO_2_-based metadevice: (**a**) SEM image of the VO_2_-based metagrating device with two outcoupling gratings at both sides; (**b**) An illustration of the metadevice with a symmetric nanoantenna and the initiated SPPs at both sides; (**c**) Schematic configuration of the VO_2_-based metadevice with a packed PCM between two Au metals and spacer layer of Al_2_O_3_; (**d**) Reflection spectra with different electrical currents to tune the spectral resonances. (**e**) Rewritable memory effect of the metadevice; (**f**–**i**) AIST-based metadevice: (**f**) A representation of an AIST-based color filter; (**g**) The generated colors using an AIST-integrated metafilter; (**h**) Resolution of created nanopixels on AIST using CAFM; (**i**) *I*–*V* curves of the AIST film obtained by CAFM. (**a**,**b**) are adapted from [79], with permission from © 2017 Nature Publishing Group; (**c**–**e**) are adapted from [80], with permission from © 2016 Nature Publishing Group; and (**f**–**i**) are adapted from [71], with permission from © 2016 WILEY-VCH Verlag GmbH & Co. KGaA, Weinheim.

VO_2_ can be engineered by phase transition on a subwavelength scale, so it has potential applications in various types of tunable metadevices [76]. The real part of RI changes when VO_2_ switches from the amorphous to the crystalline phase [77]. Several papers have reported VO_2_-based controllable plasmonic modulators that can be tuned by external stimuli [75,76,78]. A grating-based metasurface that exploits the phase transition property of VO_2_ has been developed as an active switch of surface plasmon polaritons (SPPs) at the telecom wavelength (Figure 10a) [79]. Illumination by of a monochromatic laser normal to the surface generated two SPPs (Figure 10b) [79].

Theoretical and experimental results confirmed that thermal stimuli can induce active and reversible switching of SPPs. The scattering direction of the nanograting was switched depending on the applied temperature; this phenomenon may cause the phase-transition behavior of VO_2_ [79].

Electrically triggered control has been demonstrated by imposing a VO_2_ thin film into an absorber in the MIR domain. The absorber is stimulated by Joule heating in response to electrical triggers [80]. In the metadevice structure, the VO_2_ film was sandwiched between two Au layers, with an Al_2_O_3_ layer as a spacer at the interface of Au and VO_2_ (Figure 10c). The insulating and metallic phases have different reflectance spectra under electrical currents. The optical response of the absorber was electrically adjusted; the change was thermally stable (Figure 10d). An obvious change occurred at current intensity >0.5 A, and a saturation phase occurred at current intensity >1 A [80]. The electrical characteristics of an oxide-based PCM on a photonic metadevice had electrically erasable and programmable read-only memory properties (Figure 10e). The read current was set at 0.8 A; write and erase could be achieved by adding current pulses of +0.8 A and −0.8 A, respectively. The most important advantages of this metadevice were its functionality in the MIR and its data-storage capability. Electro-thermal simulations suggested that switching speed could be increased tremendously by applying a brief high-current spike. Other studies confirmed fast switching of VO_2_-based metamaterial because of its optically determined phase evolution [81]. These results achieved efficient and switchable VO_2_-based metamadevices temporal and spatial reflection characteristics [80].

Ch-glasses are compounds of S, Se, and Te, in combination with Ge, As, Sb, and Ga. Alloy Ch-PCMs are listed based on compounds such as binary, ternary, and quaternary glass-forming PCMs (Table 2) [82]. The exceptional optical characteristics of Ch-based PCMs and their capability of changing from amorphous glass to a crystalline phase make them smart amorphous semiconductors that can be used to store information and that are suitable as tunable metasurfaces.

Light-induced responses of Chs to a photon energy close to their optical band gap, and their low-loss properties make Chs useful as basic components of photonic devices such as waveguides. Chs are important owing to their transparency over a wide range from visible to IR, and the various compositions of their stable amorphous phase [82].

The photo-induced change of atomic structure and diffusion of metals such as Ag into their arrangements cause changes in their electronic band gap, RI, and optical absorption. The photosensitivity of Chs such as As_2_S_3_ affects the properties of Ch-based thin film [83]. Chs also satisfy optical nonlinearity and IR transparency, which are required in nanophotonic devices such as waveguides [19].

Ag_3_In_4_Sb_76_Te_17_ (AIST) is another promising PCM with color-modulation range comparable to that of GST_225_ [71,84]. One proposed FP-based optical cavity is composed of a multilayer stack of two ITO layers surrounding an AIST film on a mirror (Figure 10f). The color variation under natural light was achieved by changing the AIST crystallographic phase with different ITO thicknesses (Figure 10g). Applications of AIST-based metasurfaces as nanodisplay with good resolution were confirmed by exploiting Joule heating of the PCM to achieve variation of nanopixels [71]. Two nanopixels were written by applying a reversible voltage sweep from 0 to a positive voltage, then reformed by CAFM (Figure 10h). The measured *I*–*V* curves of the AIST thin film demonstrated that change in the applied voltage yielded reliable and repeatable current switching (10i). AIST is a suitable PCM to replace GST_225_ in an active metadevice, especially for color modulation [71].

Indium antimonide (InSb) is another chalcogenide-based PCM, which has been used in a super-resolution near-field structure. InSb-based plasmonic nanoantennas with the same structure as GST-based PCM (Figure 5a,b) have been reported [12]. Compared to GST_326_, InSb has the same features and therefore has possible applications in the nonvolatile and reconfigurable resonance switching of plasmonic nanoantennas [12].

## 4. Applications and Limitations

Ch PCMs are transparent in the MIR spectra because of the low vibrational energies of their covalent bonds; as a result, Ch PCMs are suitable for use as nonlinear optics, waveguides, and hollow-core photonic fibers. They may also have other applications, such as thermal imaging due to their low T_g_ and optical signal processing due to their high glass densities, which can produce a high RI [19].

Photovoltaic IR imaging and optical/chemical sensing are potential applications of crystalline Ch PCMs. The most common implementations of Ch-based devices include all-optical data processing, optical sampling, integrated interferometry, and wavelength conversion [19]. Due to the large differences in the electrical and optical properties of the amorphous and crystalline phases of GST, it is a switchable material with tremendous applicability in non-volatile memories [15], flexible displays, rewritable optical disk storage, and computing [10].

GST is suitable for IR range and waveguides, but because of the covalent bonding of heavy elements and weak interatomic bonds, the bandgap may red-shift to the NIR and visible ranges; therefore, GST is not suitable for optical devices with high intensity of light. Their utilization for real application is questionable, and their stability as optical products under strong IR lights or as all-optical signal processors is debatable. Moreover, impurities in GST absorb light, so it may not be applicable as an optical fiber for telecommunication. To increase the IR transparency, some methods such as vacuum annealing (<130 °C to prevent roughness) and dynamic pyrolysis have been suggested. Although these techniques decrease the amount of impurities, the remaining optical losses are 2 dB·m^−1^, which is too high [19].

The key remaining impediment to use GST in metadevices is to find a composition that has the required stability, sufficient transparency, and low power consumption when melting the crystalline phase for reversible operation. Although the most crucial application of GST is in data processing and data transmission, data transfer speed is too low for practical purposes [23].

## 5. Conclusions and Outlook

We have reviewed tunable metadevices and current research based on GST metamaterials, and have discussed their possible applications in active metasurfaces and reconfigurable metadevices. We presented state-of-the-art investigations regarding tunable metasurfaces, including grating-based nanostructures, nanoantennas, and split-ring resonators. We also introduced reconfigurable metadevices such as switchable perfect absorbers, multi-functional absorbers-modulators, meta-switches, and color generating displays.

We discussed the heat generation and temperature distribution of GST-based metadevices in response to Joule heating. Finally, we discussed other proposed PCMs including VO_2_, InSb, and AIST with their applications in nanophotonics. We believe that the development of metadevices will provide methods to develop superior imaging techniques in other applications such as meteorology and geoscience. We also envision an improvement in tunable metasurfaces for use in multifunctional optoelectronic circuits, flexible integrated memories based on metasurfaces, and hybrid phase-change metadevices. Integrating the PCMs in metasurfaces is a plausible method to create compact and active electro-optic devices.

If the remaining problems can be solved, super-fast and low-dimension GST-based metadevices could be utilized in smart glasses, smart contact lenses, and synthetic retinas.

## Figures and Tables

**Table 1:** Various compositions of the germanium-antimony-telluride (GST) material with an abbreviation.

Compositions	Abbreviation
Ge_1_Sb_2_Te_4_	GST_124_
Ge_1_Sb_1_Te_2_	GST_112_
Ge_2_Sb_2_Te_5_	GST_225_
Ge_2_Sb_1_Te_4_	GST_214_
Ge_3_Sb_4_Te_8_	GST_348_
Ge_3_Sb_2_Te_6_	GST_326_
Ge_8_Sb_2_Te_11_	GST_8211_

**Table 2:** Classification of chalcogenides with their (**a**) various compounds and (**b**) different elemental compositions.

(**a**)		
**Binary**	**Ternary**	**Quaternary**
GaSb	Ga_2_Sb_2_Te_5_	Te_81_Ge_15_Sb_2_S_2_
InSb	InSbTe	AgInSbTe
InSe	GaLaS	
Sb_2_Te_3_	Ge_3_Sb_4_Te_8_ Ge_3_Sb_2_Te_6_ Ge_8_Sb_2_Te_11_	
(**b**)		
**Compositions**		**Chalcogenide**
Group V-VI		Ag_2_S_3_
		P_2_Se
Group IV-V		SiSe_2_ GeS_2_ Ge_2_Sb_2_Te_5_ GaLaS
Group III-VI		Ba_4_S_3_, InSe
Metal-Chalcogenide		MoS_3_ WS_3_ Ag_2_S-GeS_2_
